# Inter-individual differences in baseline dynamic functional connectivity are linked to cognitive aftereffects of tDCS

**DOI:** 10.1038/s41598-022-25016-5

**Published:** 2022-12-01

**Authors:** Monika Pupíková, Patrik Šimko, Martin Lamoš, Martin Gajdoš, Irena Rektorová

**Affiliations:** 1 Applied Neuroscience Research Group, Central European Institute of Technology – CEITEC, Masaryk University, Brno, Czech Republic; 2 First Department of Neurology, St. Anne’s University Hospital and Faculty of Medicine, Masaryk University, Brno, Czech Republic; 3 Brain and Mind Research Program, Central European Institute of Technology - CEITEC, Masaryk university, Brno, Czech Republic; 4 Multimodal and Functional Neuroimaging Research Group, Central European Institute of Technology – CEITEC, Masaryk University, Brno, Czech Republic; 5 International Clinical Research Center, ICRC, St Anne’s University Hospital and Faculty of Medicine, Brno, Czech Republic

**Keywords:** Neuroscience, Cognitive ageing, Cognitive neuroscience

## Abstract

Transcranial direct current stimulation (tDCS) has the potential to modulate cognitive training in healthy aging; however, results from various studies have been inconsistent. We hypothesized that inter-individual differences in baseline brain state may contribute to the varied results. We aimed to explore whether baseline resting-state dynamic functional connectivity (rs-dFC) and/or conventional resting-state static functional connectivity (rs-sFC) may be related to the magnitude of cognitive aftereffects of tDCS. To achieve this aim, we used data from our double-blind randomized sham-controlled cross-over tDCS trial in 25 healthy seniors in which bifrontal tDCS combined with cognitive training had induced significant behavioral aftereffects. We performed a backward regression analysis including rs-sFC/rs-dFC measures to explain the variability in the magnitude of tDCS-induced improvements in visual object-matching task (VOMT) accuracy. Rs-dFC analysis revealed four rs-dFC states. The occurrence rate of a rs-dFC state 4, characterized by a high correlation between the left fronto-parietal control network and the language network, was significantly associated with tDCS-induced VOMT accuracy changes. The rs-sFC measure was not significantly associated with the cognitive outcome. We show that flexibility of the brain state representing readiness for top-down control of object identification implicated in the studied task is linked to the tDCS-enhanced task accuracy.

## Introduction

The proportion of older adults in the population is increasing worldwide and the age-associated cognitive decline represents an emerging problem that society will face in the near future. While various developments have led to longer life expectancy, the proportion of the healthy years remains rather constant^1^. The economic value of a slowdown in the rate of aging that would improve health and lead to a 1-year increase in life expectancy has been estimated to be worth US$38 trillion; a 10-year increase would be worth US$367 trillion in the United States^2^. Performance decline with advancing age can be seen across a wide range of cognitive functions^3,4^; some are more affected than others. Working memory has been identified as a core cognitive function deteriorating with age that mediates age-related variance on a broad array of cognitive behaviors^5^. In order to offset the trajectory of cognitive decline in the aging population, various non-pharmacological interventions have been proposed to strengthen the cognitive functions that are sensitive to healthy or pathological aging^6–12^, including cognitive training, physical therapy, and non-invasive brain stimulation techniques. However, the effectiveness in maintaining cognitive functions varies greatly both across and within intervention types^13^.

Transcranial direct-current stimulation (tDCS) was proposed as an inexpensive and easily administered method for experimental use, and potentially also for clinical use, in an attempt to modulate cognitive functions^14^. Research aiming to modulate cognition using tDCS suggests that ongoing brain processing can be tuned and reorganized on the level of large-scale brain networks^15,16^, resulting in a favorable behavioral aftereffect via improved inter- and intra-network communication^17–21^. tDCS applies a weak direct electric current through two electrodes placed over the scalp with the goal of modulating underlying cortical excitability^22^. It has been proposed that, due to the relatively subtle neuromodulatory effect, the enhancing potential of tDCS may be more pronounced when brain networks are already engaged in cognitive tasks^15^. tDCS, in combination with cognitive training, proved its functionality in cognitive augmentation in a senior population^6,9,20,23,24^. However, accumulating evidence indicates large inter-individual variability in responsiveness to tDCS^25,26^. One-treatment-suits-all strategies have been widely adopted. As responders and non-responders to specific treatment options introduce heterogeneity into the data and lower the overall effect sizes^27,28^, study results have been inconsistent and sometimes contradictory^29–32^.

The inter-individual variability may have multiple causes; these causes are thus far poorly understood. tDCS-induced effects interact with individual differences based on demographic variables such as age, education, and sex^33–36^. Other identified factors include baseline performance on the same or similar tasks^6,37,38^, temporal spacing of sessions^34^, brain morphology^39^, and genetics^40,41^. The implementation of biomarkers in tDCS-responder identification should be encouraged, such as combinations with neuroimaging and electrophysiological methods, to increase the effectiveness of tDCS in well-selected candidates who might best profit from tDCS^42^. In particular, the study by Cerreta et al.^43^ shows the association of inter-individual variability of multi-session tDCS-induced changes with a brain state measured by resting-state functional connectivity within large-scale brain networks. However, the study did not find any associations for immediate single-session aftereffects.

Recent studies have challenged the conventional resting-state static functional connectivity (rs-sFC) analysis with its assumption of invariant rs-networks through the entire fMRI duration^44^. As the brain is an inherently dynamic system, another non-mutually exclusive approach of resting-state dynamic functional connectivity (rs-dFC) accounts for the presence of temporal variability in the resting-state functional connectivity. Individuals switch between different whole-brain connectivity profiles (often called “states”) characterized by distinct recurring functional connectivity patterns that are common for the studied population^45^. The sliding window approach is one of the most widely used methods to track time-varying functional connectivity in fMRI. The connectivity metric (Pearson correlation coefficient here) is calculated in a short-term interval window that moves along the signals in defined steps. This forms a series of correlation matrices in which connectivity states are then assessed by their clustering. For each state, higher-order summary metrics (e.g., state occurrence, dwell times, state coverage) can be estimated. Even though the debate is extensive and ongoing regarding the interpretation, functional significance, and origin of the rs-dFC and states^44,46,47^, recent advances have provided evidence for a physiological basis of rs-dFC, e.g., by combining EEG and rs-fMRI recordings^48,49^. Temporal variability was shown to reflect changes in neural activity related to cognitive, behavioral, and sensorimotor operations^50–53^. A strong correspondence was demonstrated between changing states as revealed by dynamic functional connectivity and ongoing experimentally induced cognitive states^54,55^. Researchers also examined dynamic functional connectivity during tasks and found a direct link between cognitive performance and the dynamic reorganization of the network structure of the brain^56^. The authors showed that enhanced communication between specialist regions of the brain that would otherwise remain segregated had increased an individuals ability to accomplish complex cognitive tasks. Some parameters of the dynamic system were identified as potential aspirants for sensitive markers of mental conditions which might be complementary to metrics about static brain characteristics^57^. Preliminary research has revealed alterations in specific rs-dFC features distinguishing between controls and MCI subjects^58^ or Alzheimer’s disease patients^59^. One such feature, the occurrence rate of a state, indicates how often that particular state is visited in relation to duration and has been linked to distinct brain network flexibility^44,45^ and cognitive flexibility^60^.

Working memory, i.e., the ability to adaptively maintain and simultaneously manipulate information^61,62^ to be employed in ongoing processing, is related to higher-order cognitive skills such as multitasking and learning^63^. It is therefore central to the execution of a variety of daily functions^64^. Major cognitive brain networks, such as the frontoparietal control (FPCN) and dorsal attention networks (DAN), are important in governing working memory processes^65,66^. A significant number of studies have investigated age-related modifications in functional networks using static functional connectivity and have revealed disruptions/reorganizations within certain functional brain networks^67–69^, including the FPN and the DAN. In addition, the default-mode network (DMN), which is typically activated during internally focused cognitive processes^70^ and suppressed during the performance of externally directed tasks^71^, is characterized by patterns of age-related intra-network decrease and increased between-network connectivity^72–74^. This shift from intra-network to more pronounced inter-network connectivity seems to be global among brain networks in healthy aging^75^, referred to as reduced network segregation and increased integration. Age-related altered interplay between task-positive and task-negative networks has been associated with compromised working memory performance^76,77^. However, working memory relies on the ability to engage diverse cognitive control systems^78,79^ and thus on dynamic and flexible coordination across multiple large-scale brain networks that transiently link distributed brain regions in response to changing task demands^80–82^. While it is generally assumed that cognitive deficits in older adults are related to reduced brain flexibility, these might not be appropriately addressed by rs-sFC, due to its nature. It has been suggested that the study of rs-dFC can unveil flexibility in the functional coordination between different sub-networks and provide a deeper understanding of distinct brain changes with aging^83^. A recent study revealed that reduced brain flexibility in a senior population due to disruptions in brain state dynamics was associated with discrete cognitive deficits during a working memory task that became more pronounced with advancing age^84^. Further, the ease of state transitions from one state to another and occurrences of some particular states decreased with advancing age^83,85,86^. These alterations in brain dynamics due to aging were associated with cognitive performance^85^. Age-related changes associated with brain network dynamic flexibility may provide potential markers of risk for, and resilience to, age-related cognitive decline across the lifespan^46^. We tested whether the occurrence of distinct brain states at rest predicts the magnitude of cognition-targeted intervention aftereffects, such as tDCS coupled with cognitive training.

Our aim was to explore whether inter-individual differences in brain state dynamics of healthy seniors, as evaluated by rs-dFC data analysis, might influence the magnitude of tDCS-induced cognitive aftereffects. We also aimed to compare rs-dFC and conventional rs-sFC methods to determine which one is more precisely related to specific immediate stimulation-induced aftereffects. To achieve our aims, we utilized data from our previous study of bifrontal tDCS coupled with cognitive training in healthy older adults^20^. The data had indicated a significant stimulation effect upon cognition. The original study was conducted in a double-blind, cross-over design aimed at enhancing performance in a visual object matching task (VOMT). The bifrontal montage, with anode over the left dorsolateral prefrontal cortex (lDLPFC), significantly enhanced VOMT accuracy as compared to the sham stimulation. This was accompanied by a significant stimulation × time interaction in rs-sFC, measured by the magnitude of resting-state functional connectivity between the stimulated seed and the fronto-parietal control network (FPCN). We now computed a regression model to explore whether baseline resting-state functional connectivity could contribute to variability in tDCS-induced immediate cognitive aftereffects, encompassing both rs-sFC and rs-dFC measures.

## Methods

### Sample

A cohort of healthy seniors, all at least 60 years of age, were enrolled in the study. Only participants with no serious neuropsychiatric conditions and with intact cognition were included in the experiments on the basis of a complex neuropsychological examination prior to the study (for details see^20^); no participants had ferromagnetic metals in their bodies (due to the presence of MRI data acquisition). Demographic data included age, sex, and education. Each subject signed the informed consent form in accordance with the ethics codes and relevant regulations approved by the ethics committee of Masaryk University.

### Study design and procedure

**Figure 1:**
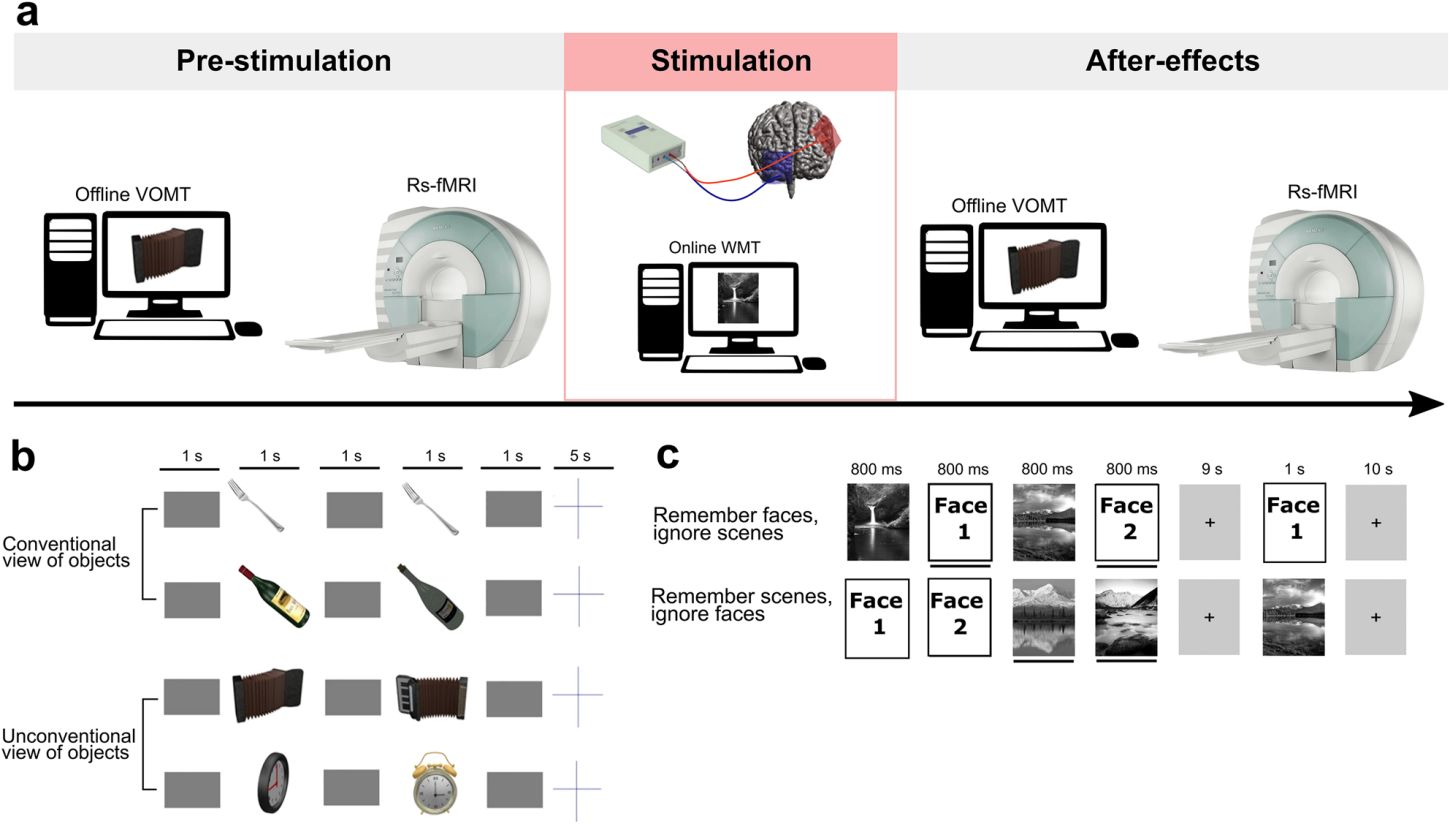
Experimental design and methods. (**a**) The crossover design involved two sessions with real 2 mA stimulation/ sham tDCS with a concurrent working memory task. Prior to and after the stimulation, participants performed a visual object matching task (VOMT) and underwent resting-state fMRI. (**b**) Offline VOMT—subjects respond whether the two consecutive objects are the same or different by pressing a YES/NO button in two difficulty levels (conventional view of objects—lower difficulty level; unconventional view of objects—higher difficulty level). (**c**) Online WMT—subjects view a block of faces and scenes (2 + 2, randomized order) preceded by a specific command on how to react to a probe that follows each block. Subjects respond whether the probe is consistent/ inconsistent with the prior instruction by pressing a YES/NO button. Freely available face photographs from Chicago^87^ and Glasgow^88^ face databases were used as a face stimuli in the task.

Subjects participated in a double-blind crossover design study as described previously^20^. All participants underwent a series of four tDCS stimulations using two distinct electrode montages (bi-frontal/right fronto-parietal), with corresponding sham stimulation over the same stimulation areas (see Fig. 1). Data from the bi-frontal montage was used for the current study. All participants had fMRI prior to and immediately after tDCS in each experimental session. The tDCS and the VOMT offline task were performed in the NIBS laboratory placed next to the MRI scanner and it took less than 5 min to move subjects between the two laboratories. The main behavioral outcome, VOMT was performed before and after the tDCS with a visual working memory task (WMT) with faces and scenes as an “online” cognitive training task during the tDCS stimulation (online WMT). Both tasks were practiced by the participants during the baseline (opening) session to prevent high learning effects between the first and the second stimulation session. Prior to the experimental sessions, each subject underwent T1 MRI sequence scanning to enable individual targeting of tDCS montage (for further details see below). The study was approved by The Masaryk University Research Ethics Committee. The study was carried out in accordance with relevant guidelines and regulations. The trial was preregistered in ClinicalTrials.gov under NCT04134195.

### tDCS

tDCS was performed through a battery-driven stimulator (DC-Stimulator Plus, NeuroConn GmbH, Germany). The anode was placed over the lDLPFC (MNI − 40 32 30) and the cathode over the right middle frontal gyrus (MFG; MNI 44 40 − 10) in the bifrontal stimulation protocol^20^. We used the T1 MRI scan-based frameless stereotactic neuro-navigation targeting with Brainsight 2, to specify the exact location of the electrode center in each individual. A current of 2 mA was delivered using two rubber electrodes (5 × 5 cm) for 20 min, with initial ramp-up and final ramp-down phases of 30 s. The electrode was held in place by conductive paste (Ten20 Conductive Paste gel, Weaver and Company). The sham stimulation was applied with the same settings, but the stimulator was turned off after 30 s. The impedance was controlled by the device throughout the session; an excess of limits would have led to an automatic termination of stimulation.

### Behavioral tasks

Throughout the study, we used two different visual WMT. VOMT was our main behavioral outcome. The task consists of multiple successive paired images of common objects. The second image of each pair is either the same or different from the first image (different object identity or object orientation). Participants are instructed to respond as quickly as possible by pressing a YES button if the second object of the paired images is the same as the first object (regardless of spatial orientation) or by pressing a NO button if the second object is different. We collected the number of correct responses and reaction times (RT) of both conditions—conventional view (lower difficulty level) condition and unconventional view (higher difficulty level) condition with rotated object views. We used different versions of the task for every session, balanced in difficulty. The main outcome was overall accuracy (the percentage of the correct responses) based on our previous study^20^.

The online WMT, adapted from Gazzaley et al.^78^, involved faces and outdoor scenes to be remembered which was performed during the stimulation session (active, sham). The task consisted of two subtasks in which aspects of visual information were kept constant while the target instruction changed. Subjects viewed a block of faces and scenes (2 + 2, randomized order) preceded by a specific command on how to react to a probe that followed each block. Subjects responded to whether the probe was consistent/ inconsistent with the prior instruction by pressing a YES/NO button. The number of correct responses and reaction times (RT) were collected (for more details see^20^).

### MRI data acquisition and pre-processing

We acquired MRI data with a 3.0 T Magnetom Siemens Prisma. Our MR protocol involved a T1 MPRAGE sequence (TR 1620 ms; TE 2.44 ms; voxel size 1 × 1 × 1 mm; FoV 256 × 256 mm; flip angle 8°; 224 transversal slices) and two sessions of resting-state fMRI (rs-fMRI; n = 25; TR 850 ms; TE 35.2 ms; voxel size 2 × 2 × 2 mm; FoV 208 mm; flip angle 45°; 80 transversal slices; 700 scans; multiband factor 8; and overall duration of resting-state acquisition 9.5 min).

The data was checked for spatial abnormalities using the tool mask explorer^89^. We controlled for the excessive presence of movement in the data using framewise displacement (FD)^90^; we excluded all datasets exceeding the condition FD < 0.5 mm in less than 20% of scans (7 subjects excluded) as used e.g. in^20^. The data was pre-processed in SPM12 running under MATLAB R2019a, using realign and unwarp, spatial normalization, and spatial smoothing (FWHM 5 mm).

### Independent component analysis (ICA)

We decomposed pre-processed BOLD rs-fMRI data on statistically independent components to identify resting-state brain networks. We used the toolbox GIFT (https://trendscenter.org/software/gift/;^91,92^). The ICA was performed with the INFOMAX algorithm and GICA back-reconstruction algorithm. The reliability of the components was determined with the ICASSO toolbox^93^. The optimal number of components was based on the minimum description length criterion^94^. In our data, 30 stable components were estimated, out of which we identified 10 brain networks.

### Dynamic functional connectivity

Sliding window correlations, i.e. Pearson’s approach^95,96^, between the temporal-series of 10 selected independent components (ICs) were calculated. For each subject and session, a window length of 60 s and 90% overlap formed a series of 89 correlation matrices 10 × 10. The mean correlation matrix was subtracted from each matrix. The series of de-meaned matrices from each subject and session were concatenated across third (temporal) dimension.

K-means clustering applied on concatenated matrices was used to find re-occurring functional network states^49^. The optimal number of clusters was determined by the mean criterion, which contained measures of Calinski-Harabasz index, Davies-Bouldin index, and silhouette values. The clustering algorithm was repeated 1000 × with random initialization of centroid positions. The final cluster centroids represent functional network states. State vectors, composed as the assignment of each subject correlation matrix in a time-series to the nearest cluster by a k-means algorithm, were used to extract parameters of the state dynamics^97^. These included time coverage, i.e. the percentage of data covered by a specific state, and occurrence i.e. the number of state segments divided by duration. The higher the parameter value, the more often the state appears in a shorter duration. Higher state occurrence has been interpreted as higher cognitive flexibility^98^.

### Static functional connectivity

For the rs-sFC, a magnitude of rs-connectivity between the lDLPFC and left inferior parietal lobule as part of the FPCN was used in the regression model as an explanatory variable based on our previous results (lDLPFC-FPCN;^20^). We computed rs-sFC as follows: first, we used ICA on resting-state functional connectivity data and manually identified spatial components representing the FPCN. Local maxima were chosen as regions of interest. Next, Pearson correlations between representative signals, converted to z values using the Fisher r-to-z transformation, were computed across the whole duration of a resting-state scan. Our focus was on the rs-sFC between stimulation seeds (seeds placed on the area underneath the anode electrode center) and the network seeds of FPCN (for further details see^15^).

### Statistical analyses

For each stimulation session, the change in performance scores was defined as VOMT overall accuracy at post tDCS minus the VOMT overall accuracy at pre tDCS. Thus, a positive value of the change indicates an improvement in VOMT overall accuracy. In the current study, we aimed to investigate the association between this dependent variable and rs-dFC/ rs-sFC measures at baseline.

First, we inspected the distribution of the behavioral and fMRI variables. Missing values were computed if other related variables for the case were available (1 value in the whole dataset). Non-normal distributions were log-transformed to meet normal distribution. At the baseline, bi-variate Pearson correlations were performed to identify significant relationships with baseline performance in VOMT accuracy and rs-sFC, rs-dFC, or demographic variables. Backward regression analysis was then performed (removal criteria: *p* ≥ 0.10) to find the best variable to explain the variability in the magnitude of tDCS-induced improvements in VOMT accuracy. The outcome variable for the model was the pre-post difference in the overall accuracy of VOMT. Independent factors (predictors) included the occurrence rate of identified rs-dFC states 1–4 (a separate variable for each state) and a magnitude of lDLPFC-FPCN rs-sFC. If the regression model was significant, we then conducted sensitivity analyses by using a second regression model that tested whether the model remained significant following the inclusion of additional variables in the model shown to be associated with tDCS effects in previous literature: demographic variables (age, sex, education) and baseline performance^37^ using forced entry multiple regression analyses. Before performing multiple regressions, independent variables were tested for multicollinearity (i.e., strong correlations among predictor variables, Pearson correlation coefficient (r) greater than 0.7) and homoscedasticity. Results were considered significant at *p* < 0.05. As this was an exploratory study aiming to identify potential candidates for further confirmatory trials, we did not correct for multiple comparisons. We emphasize that any significant results should not be interpreted as confirmatory, but rather exploratory for future hypothesis-driven trials.

## Results

### Subjects

Twenty-five healthy seniors (68.84 ± 4.65 years old; 17/8 women/men ratio; all Caucasians) completed the study. All participants had a high school or higher education level of 14.48 ± 2.64 years. Seven subjects were excluded due to low fMRI data quality (the excessive presence of participant movement in the scanner), one subject was excluded due to extreme values of rs-dFC measures.

### Independent component analysis

**Figure 2:**
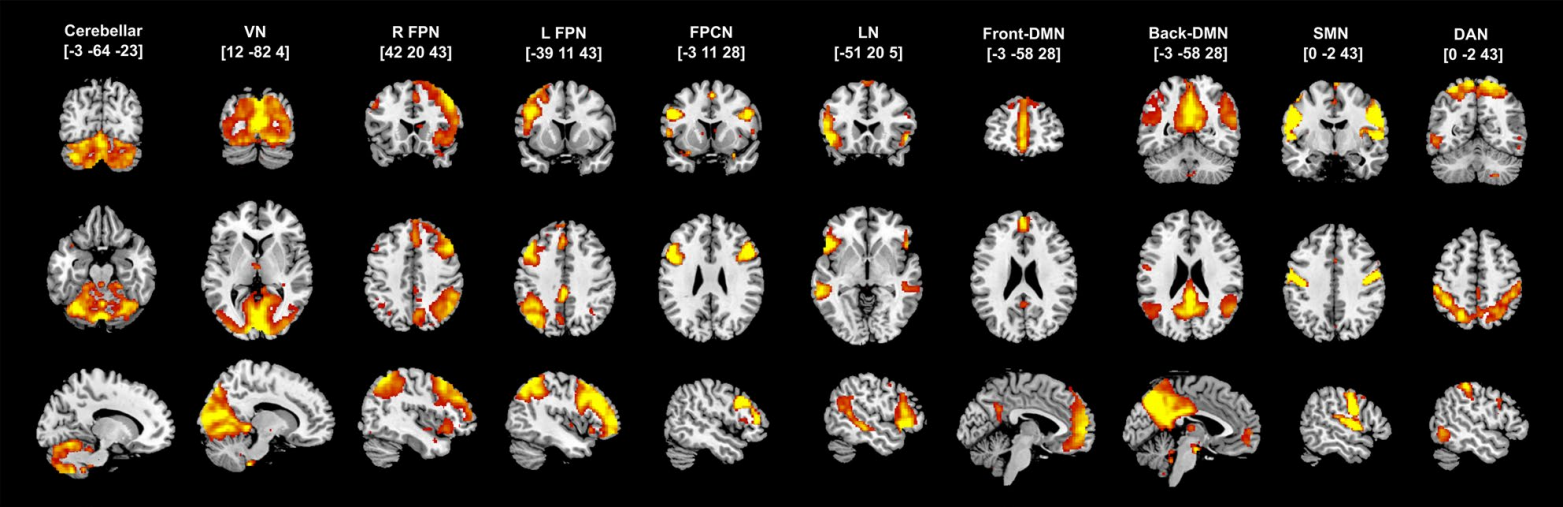
Ten ICA components utilized for the rs-dFC analysis.

We selected 10 components that represent functional networks and have been widely identified and reported in the literature by others^99–103^: cerebellar network, front and back default mode network (front/back-DMN), visual network (VN), right and left frontoparietal network (r/l-FPN), frontotemporal (language) network (LN), dorsal attentional network (DAN), frontoparietal control network (FPCN), and sensorimotor network (SMN; Fig. 2).

### Dynamic functional connectivity states

**Figure 3:**
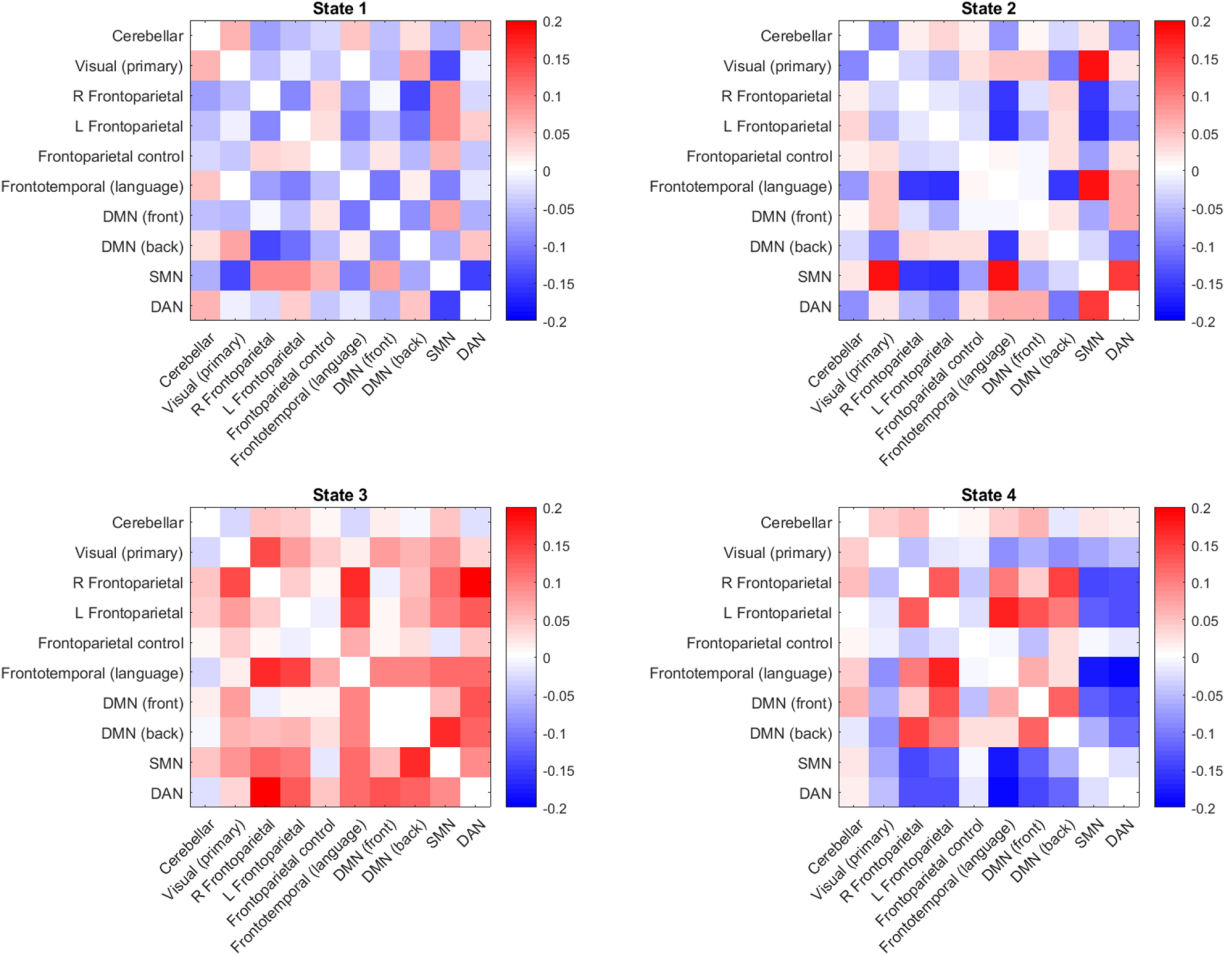
Four identified rs-dFC states (1–4 from the upper left to lower right). Each matrix depicts mutual correlations between each component identified using the ICA. Dark blue suggests a high negative correlation, and dark red suggests a high positive correlation. Note: *DMN* default-mode network, *SMN* sensorimotor network, *DAN* dorsal attentional network.

The analysis identified four rs-dFC states (Fig. 3). In brief, state 1 is a sparsely connected dynamic functional state, the posterior part of the DMN is clearly anticorrelated (i.e., network fluctuations are in the opposite direction) with the right FPN, and the SMN is anticorrelated with both DAN and primary VN, thus representing the “true” resting state in terms of both cognitive and motor activity. In state 2, both left and right FPN are anticorrelated with the language and SMN networks. The posterior part of the DMN is particularly anticorrelated to the language network. At the same time, the SMN is highly interconnected with the visual, DAN, and language networks. This state may represent readiness for processing tools and objects, as indicated in the “action reappraisal” concept. This concept suggests that object knowledge is constituted by information inscribed within the motor and sensory systems thus stressing the automatic lower-level processing of information^104^. State 3 is a hyperconnected state which reveals particularly tight connection between the right FPN and DAN, visual and language networks. The (posterior) DMN is highly correlated with the SMN. The state may thus represent readiness for both motor and visual attention reaction to salient stimuli. In state 4, the left FPN (where the tDCS anode was positioned) is highly correlated with the language network which is in turn anticorrelated with the SMN and DAN. This state may represent readiness for top-down control of object identification with an involvement of temporal regions of the ventral visual pathway, and covert verbalization/naming of an identified object with an engagement of semantic language processing.

### Baseline rs-sFC and rs-dFC measures

The coverage of each state varied between 19% (state 4) to 35% (state 1), see Supplementary Table S1. For the occurrence of each state, see Table 1. Coverage and occurrence of rs-dFC states and a rs-sFC measure—lDLPFC-FPCN connectivity did not differ at baseline between real and sham stimulation conditions (*p* > 0.05, data not shown).

**Table 1:** Mean rs-dFC outcomes.

Variable	Mean	Std. deviation
Occurrences of a state 1	0.174	0.074
Occurrences of a state 2	0.220	0.121
Occurrences of a state 3	0.228	0.105
Occurrences of a state 4	0.223	0.133

### Regression model

At baseline, there were no significant correlations with the baseline VOMT accuracy performance and rs-sFC, rs-dFC or demographic variables. All assumptions for multiple regression were met. Backward regressions showed that for active stimulation, the occurrence of state 4 was the only significant variable explaining the variability in overall accuracy change (*R*^2^ = 0.304; F_(1,16)_ = 6.555; *p* = 0.022). The final model of a backward regression is shown in Table 2. The *R*^2^ change between models varied between 0.000 and 0.026 (for the stepwise report of the backward regression see Supplementary Table S3). The model showed that the higher the occurrence of state 4 at baseline, the higher change of overall VOMT accuracy (β = 0.551). The occurrence rate of states 1 to 3 and the rs-sFC measure were not significantly associated with the accuracy change. State 4 remained significant (*p* = 0.046) even after correcting the model for demographic variables and baseline performance; however, the overall model was not significant (*R*^2^ = 0.378; F_(1,16)_ = 1.334; *p* = 0.320, see Supplementary Table S4). No significant associations were found for the sham condition.

**Table 2:** The final regression model statistics.

Variable	B	95% CI for B		Std. β	t	*p*	*R*^2^	Std. residual	
		Lower bound	Upper bound					Mean	SD
Occurrences of state 4	0.125	0.021	0.230	0.551	2.560	**0.022**	0,304	0.000	0.968

Significance values are in bold.

## Discussion

In this study, we investigated whether baseline resting-state functional connectivity might contribute to individual differences in the tDCS-induced VOMT effects in healthy older adults with a novel insight from functional connectivity temporal dynamics. The flexibility in which different rs-dFC patterns, i.e. brain states, are visited seems to be crucial for efficient and adaptable communication within the brain^105,106^. Brain state occurrence rate (i.e. our rs-dFC parameter of interest) has been reported to reflect cognitive flexibility^59,60,98^. In line with the previous literature, we were able to identify several distinct sparsely, densely, and intermediately connected rs-dFC patterns, similar to those reported in other studies^45,59,85,97,107^.

In line with previous literature^45,59,107^, participants spent most of the time in the sparely connected rs-dFC state (state 1 in this study) with relatively weak connections—mostly weak anti-correlations (or correlations near zero) between the task-negative and task-positive networks. This state may thus represent a “true” resting state in terms of both cognitive and motor activity. The frequency of a similar state was linked to the amount of self-focused thoughts^108^. The state has been suggested to reflect a “ground state”, a preferable state of the brain with lower information transfer while preserving the maximum of energy saved^109^. The states with weak and moderate correlations (correlations near zero) between brain networks were also considered as “metastable” brain states that avoid extreme brain configurations, allowing for the flexible reconfiguration of neural networks^110^. On the other hand, other states have been previously interpreted as reflecting temporary deviations arising due to cognition, readiness to react to internal/ external stimuli, or due to other neurophysiological processes^107^. We demonstrate that participants with a pre-existing higher occurrence rate of a particular rs-dFC state showed better cognitive response to tDCS stimulation. Notably, this rs-dFC state (state 4) reveals increased connection of the networks that are known to be engaged in a cognitively demanding visual working memory control. The total coverage of state 4 was lower than that of state 1. In more detail, the left FPN (where the tDCS anode was positioned) was highly correlated with the fronto-temporal network with a predominant left-sided involvement. This network is also referred to as the language network^111^. Therefore, we may speculate that this state represents readiness for top-down control of object identification in the VOMT, which includes covert naming of an object and involves semantic language processing^112–114^. As the presentation of objects is ambiguous in the VOMT, object identification is required, thus processes beyond the basic perceptual comparison relying on visual networks are necessary. The ventral temporal lobe—specifically Broadmann area 37, partly covered in our fronto-temporal language network—is a multimodal language region in the ventral visual pathway that was shown to process semantic information about an object^115,116^. Moreover, its intact connections to the middle temporal gyrus (which was substantially covered by the language network) are necessary for intact visual object recognition^117,118^, particularly related to object meaning and knowledge^119–121^.

Participants who had a higher occurrence of this tightly connected rs-dFC state 4 before the stimulation improved more than those with a lower occurrence of the state. Previous literature showed that patients with cognitive dysfunctions (Alzheimer’s disease; dementia with Lewy bodies, DLB) spend more time in the lower inter-network connectivity state (i.e. energy saving mode) and switch less often into more highly and specifically connected network configurations^59^. Another study from the same group reported marked and generalized slowing of the network dynamics in a DLB cohort in comparison with healthy participants^98^. In the same vein, more pronounced brain flexibility as measured by the temporal variability of functional connectivity in healthy individuals, has been shown to be related to superior performance on a range of cognitive tests across different domains (e.g. alertness, memory^106^). It seems that the efficiency of a healthy brain allows for a balance between metabolic expenditure and readiness for a more specific response to situational demands^122,123^. Notably, state 4 also involved a high correlation between the frontoparietal and DMN networks, which was shown to be a central feature of neurocognitive aging as revealed by rs-sFC, termed the “default-executive coupling hypothesis of aging”^72,124^. A similar state (in terms of co-activation among the FPCN and DMN) and reduced flexibility, e.g., longer dwell times, was related with reduced cognitive performance in healthy aging^85^. We believe that our data provide support for the phenomenon of “the rich get richer” observed in previous studies^33,37^, in which performance improvement is more expressed in a subset of high-performers or in people with higher education. In our study, individuals with higher flexibility of state 4 showed better responsiveness to tDCS. However, this interpretation must be treated cautiously. Our results do not confirm any causal relationship but only an association between specific brain rs-dFC and tDCS-induced cognitive aftereffects as they stem from the temporal dynamics of identified correlation matrices.

In contrast to results found in the literature^33,37^, neither education nor baseline performance could explain the variability in tDCS-induced effects in our sample when forced into the model. However, all subjects in this study were high-performers with at least a high school education (14.58 mean years of education in our study, as compared to 13.5 years of education in the lower educated group in^37^; therefore, the variability in these parameters was rather low.

In our regression model, we also considered the strength of rs-sFC between the stimulated seed (lDLPFC) and FPCN, which had shown a significant stimulation × time interaction after tDCS in our original study^20^. Unlike rs-dFC measures, the magnitude of baseline rs-sFC was not associated with stimulation-related outcome. This finding is in accordance with the results of a previous study by Cerreta et al.^43^ in which the authors reported that rs-sFC of the DMN or FPCN could not predict tDCS-induced response in a 2-back WM task accuracy after a single session stimulation with the anode placed over the right DLPFC. We hypothesize that using rs-dFC as compared to rs-sFC measures may be necessary to identify tDCS responders. Future research is warranted to confirm this hypothesis.

We are aware of some limitations of this study. The study sample included only participants with more than 12 years of education, thus our sample showed smaller variability in task accuracy, underrepresenting low performers and limiting the transferability to the general population of older adults. In this exploratory study we did not use multiple comparison correction in regression models.

In conclusion, this study demonstrates that the brain state as measured by rs-dFC plays a role in inter-individual differences in tDCS-induced immediate cognitive aftereffects and that the relationship between the dynamics of a particular rs-dFC and stimulation-induced aftereffects is specific in terms of networks engagement and the anode position (i.e. over the lDLPFC, which is part of the left FPCN that provides visual processing control for VOMT). Individuals who exhibited higher flexibility of this specific task-related state were more responsive to the bi-frontal tDCS coupled with cognitive training. We also showed that rs-dFC analysis is better correlated with the immediate tDCS response magnitude, at least in participants with > 12 years of education, as compared to a regular rs-sFC analysis.

## Supplementary Information

Supplementary Tables.

## Data Availability

The datasets generated and/or analysed during the current study are not publicly available due accordance with informed consents signed by study participants but are available from the corresponding author on reasonable request.
