# Supplementary material for: Inter-individual differences in baseline dynamic functional connectivity are linked to cognitive aftereffects of tDCS
Source: Sci Rep. 2022 Dec 1;12:20754. doi: 10.1038/s41598-022-25016-5 (PMC9715685; doi:10.1038/s41598-022-25016-5)
Supplement: Supplementary file 1 — Supplementary Tables. [file 41598_2022_25016_MOESM1_ESM.docx]

Supplementary Material

| **Demographic variables** |  | **Mean** | **SD** | **Min** | **Max** |
| --- | --- | --- | --- | --- | --- |
| Age |  | 68.84 | 4.65 | 62 | 78 |
| Education |  | 14.48 | 2.64 | 11 | 19 |
| **Cognitive variables** | **Test** | **Mean** | **SD** | **Min** | **Max** |
| Visual perception | JLO | 24.36 | 3.57 | 17 | 30 |
|  | ROCF-C | 33.58 | 2.05 | 29 | 36 |
| Memory | ROCF-I | 17.72 | 4.95 | 9 | 27 |
|  | ROCF-D | 17.14 | 5.31 | 5 | 26 |
|  | ROCF-R | 20.13 | 1.81 | 17 | 23 |
|  | WL-I | 30.2 | 3.91 | 21 | 24 |
|  | WL-D | 5.5 | 2.3 | 1 | 9 |
|  | WL-R | 22.54 | 1.35 | 8 | 22 |
| Attention, psychomotor speed | TMT-A | 39.41 | 9.64 | 24 | 70 |
|  | ST-W | 81.37 | 10.79 | 56 | 101 |
|  | ST-C | 68.41 | 10.41 | 51 | 88 |
| Executive functions | ST-CW | 37.33 | 8.33 | 24 | 58 |
|  | ST-I | 2.09 | 7.16 | -16.7 | 17 |
|  | TMT-B | 88.04 | 22.02 | 52 | 130 |
|  | VFT-S | 24.92 | 6.39 | 15 | 38 |
|  | VFT-L | 41.88 | 9.47 | 27 | 63 |
|  | FPT | 31.18 | 9.24 | 12 | 51 |
| Language | TT | 35.27 | 0.66 | 34 | 36 |
|  | BNT-30 | 27.22 | 2.02 | 23 | 30 |
| Depression | BDI II | 9.36 | 5.5 | 3 | 27 |
| Daily functioning | FAQ | 0.08 | 0.39 | 0 | 2 |

Table S1 Demographic and cognitive characteristics of the study group. JLO: Judgment of Line Orientation; ROCF-C, I, D, R: Rey-Osterrieth Complex Figure Test: copy, immediate, delayed recall, and recognition; WL-I, D, R: Wechsler Memory Scale III: Word List immediate, delayed recall, and recognition; TMT-A: Trail-Making Test part A; ST-W, C, CW, INT: Stroop Color and Word Test, word, color, color-word, and interference score; TMT-B: Trail-Making Test part B; VFT-S, L: Verbal Fluency Test, semantic, lexical; FPT: five-point test; TT: Token Test; BNT-30: Boston naming test; BDI II: Beck’s depression inventory.

|  |  | N | Mean | Std. Deviation | t | P |
| --- | --- | --- | --- | --- | --- | --- |
| Coverage of state 1 | Real | 17 | 0.336 | 0.075 | 0.638 | 0.532 |
|  | Sham | 17 | 0.352 | 0.103 |  |  |
| Coverage of state 2 | Real | 17 | 0.223 | 0.107 | -0.509 | 0.617 |
|  | Sham | 17 | 0.218 | 0.089 |  |  |
| Coverage of state 3 | Real | 17 | 0.245 | 0.096 | -0.188 | 0.853 |
|  | Sham | 17 | 0.231 | 0.083 |  |  |
| Coverage of state 4 | Real | 17 | 0.196 | 0.074 | 0.363 | 0.721 |
|  | Sham | 17 | 0.199 | 0.078 |  |  |

Table S2 Mean differences in coverage of each state at the baseline

| Model |  | Variables | β | (SE) | Std. β | t |  | P | R^2^ | R^2^ change | p |
| --- | --- | --- | --- | --- | --- | --- | --- | --- | --- | --- | --- |
| 1 |  | Occurrences of state 1 | 0.030 | 0.222 | 0.040 | 0.134 |  | 0.896 | 0.333 | 0.333 | 0.415 |
|  |  | Occurrences of state 2 | 0.005 | 0.061 | 0.021 | 0.081 |  | 0.937 |  |  |  |
|  |  | Occurrences of state 3 | -0.047 | 0.083 | -0.150 | -0.570 |  | 0.580 |  |  |  |
|  |  | Occurrences of state 4 | 0.124 | 0.069 | 0.547 | 1.817 |  | 0.097 |  |  |  |
|  |  | Connectivity DLPFC - FPCN | 0.010 | 0.068 | 0.038 | 0.145 |  | 0.888 |  |  |  |
| 2 |  | Occurrences of state 1 | 0.029 | 0.212 | 0.039 | 0.135 |  | 0.895 | 0.332 | 0 | 0.265 |
|  |  | Occurrences of state 3 | -0.047 | 0.079 | -0.148 | -0.591 |  | 0.566 |  |  |  |
|  |  | Occurrences of state 4 | 0.124 | 0.066 | 0.547 | 1.896 |  | 0.082 |  |  |  |
|  |  | Connectivity DLPFC - FPCN | 0.011 | 0.063 | 0.044 | 0.181 |  | 0.860 |  |  |  |
| 3 |  | Occurrences of state 3 | -0.050 | 0.074 | -0.157 | -0.671 |  | 0.514 | 0.331 | -0.001 | 0.144 |
|  |  | Occurrences of state 4 | 0.120 | 0.053 | 0.526 | 2.277 |  | **0.040** |  |  |  |
|  |  | Connectivity DLPFC - FPCN | 0.010 | 0.059 | 0.038 | 0.166 |  | 0.871 |  |  |  |
| 4 |  | Occurrences of state 3 | -0.051 | 0.070 | -0.163 | -0.733 |  | 0.476 | 0.330 | -0.001 | 0.061 |
|  |  | Occurrences of state 4 | 0.119 | 0.051 | 0.523 | 2.354 |  | **0.034** |  |  |  |
| 5 |  | Occurrences of state 4 | 0.125 | 0.049 | 0.551 | 2.560 |  | **0.022** | 0.304 | -0.026 | **0.022** |

Table S3 Step-wise results of the regression analyses

| Variable | B | 95% CI for B | | Std. β | t |  | p | R^2^ | Std. Residual | |
| --- | --- | --- | --- | --- | --- | --- | --- | --- | --- | --- |
|  |  | Lower Bound | Upper Bound |  |  |  |  |  | Mean | SD |
| Occurrences of state 4 | 0.128 | 0.003 | 0.254 | 0.564 | 2.247 |  | **0.046** | 0,378 | 0.000 | 0.829 |
| Age | -0.001 | -0.008 | 0.007 | -0.057 | -0.194 |  | 0.850 |  |  |  |
| Education | -0.003 | -0.014 | 0.009 | -0.138 | -0.496 |  | 0.630 |  |  |  |
| Sex | 0.002 | -0.087 | 0.091 | 0.021 | 0.057 |  | 0.956 |  |  |  |
| Baseline overall accuracy | 0.212 | -0.516 | 0.940 | 0.251 | 0.640 |  | 0.535 |  |  |  |

Table S4 Regression model corrected for demographic variables
